# Feasibility of reduced-dose posttransplant cyclophosphamide and cotransplantation of peripheral blood stem cells and umbilical cord-derived mesenchymal stem cells for SAA

**DOI:** 10.1038/s41598-020-80531-7

**Published:** 2021-01-08

**Authors:** Yingling Zu, Jian Zhou, Yuewen Fu, Baijun Fang, Xinjian Liu, Yanli Zhang, Fengkuan Yu, Wenli Zuo, Hu Zhou, Ruirui Gui, Zhen Li, Yanyan Liu, Huifang Zhao, Chengjuan Zhang, Yongping Song

**Affiliations:** 1 Department of Hematology, Affiliated Cancer Hospital of Zhengzhou University and Henan Cancer Hospital, Zhengzhou, 450000 People’s Republic of China; 2 Center of Bio-Repository, Affiliated Cancer Hospital of Zhengzhou University and Henan Cancer Hospital, Zhengzhou, 450000 People’s Republic of China

**Keywords:** Immunology, Bone marrow transplantation, Transplant immunology

## Abstract

Posttransplant cyclophosphamide (PTCy) as graft-versus-host disease (GVHD) prophylaxis is an effective strategie for patients receiving matched sibling donor hematopoietic stem cell transplantation (MSD-HSCT) and haploidentical HSCT (haplo-HSCT). We evaluated the effectiveness and safety of reduced-dose cyclophosphamide, 20 mg/kg for 13 patients in MSD-HSCT cohort and 25 mg/kg for 22 patients in haplo-HSCT cohort, on days + 3, + 4 combined with cotransplantation of peripheral blood stem cells (PBSCs) and human umbilical cord-derived mesenchymal stem cells (UC-MSCs) for severe aplastic anemia (SAA). In MSD-PTCy cohort, the times to neutrophil and platelet engraftment were significantly shorter than those in the MSD-control cohort (P < 0.05). The cumulative incidence of acute GVHD (aGVHD) at day + 100 (15.4%) was lower than that in the MSD-control cohort (P = 0.050). No patient developed chronic GVHD (cGVHD). The 1-year overall survival (OS) and event-free survival (EFS) rates were 100% and 92.3%. In haplo-PTCy cohort, the times to neutrophil and platelet engraftment were significantly shorter than those in the haplo-control cohort (P < 0.05). The cumulative incidences of aGVHD at day + 100 and 1-year cGVHD were 31.8% and 18.2%, and the 1-year OS and EFS rates were 81.8% and 66.9%. Reduced-dose PTCy and cotransplantation of PBSCs and UC-MSCs is an acceptable alternative to patients with SAA.

## Introduction

Severe aplastic anemia (SAA), a life-threatening disease, can be cured by allogeneic hematopoietic stem cell transplantation (allo-HSCT) based on human leukocyte antigen (HLA)-appropriate donors^1^. Nevertheless, patients undergoing HSCT always suffer from related complications, including graft-versus-host disease (GVHD), graft rejection and infection, especially those lacking conventional HLA-identical donors^2^. Thus, the use of haploidentical hematopoietic stem cell transplantation (haplo-HSCT) is limited by the incidences of graft failure and GVHD^3^. Graft sources are usually bone marrow (BM) and peripheral blood stem cells (PBSCs). PBSCs are increasingly used owing to the ease of obtaining cells and the high stem cell counts. However, the use of PBSCs is related to a higher incidence of acute GVHD (aGVHD) and chronic GVHD (cGVHD) than the use of BM^4^.

Mesenchymal stem cells (MSCs) possess the abilities of potential self-renewal, multilineage differentiation and regulation of immune function. Hence, MSCs reduce the risk of aGVHD and cGVHD^5^. It has been shown that MSCs can not only facilitate the engraftment of neutrophils and platelets but also promote donor chimerism^6^. In addition, human umbilical cord-derived MSCs (UC-MSCs) have a higher proliferation and differentiation ability than BM-MSCs^7^. Therefore, UC-MSCs play a considerable role in the clinical application of allo-HSCT for patients with SAA.

Currently, strategies for reducing GVHD involve the use of antithymocyte globulin (ATG) and posttransplant cyclophosphamide (PTCy) in haplo-HSCT. However, the high cost of ATG is a major obstacle to routine application. In recent years, PTCy as a haploidentical T cell-depleting protocol has become an effective alternative strategy for patients with nonmalignant hematological disorders in the haplo-HSCT setting^8,9^. PTCy has displayed a better outcome as GVHD prophylaxis than ATG in mismatched unrelated donor (MMUD) HSCT^10^. Several studies have obtained encouraging results after evaluating the efficacy of PTCy at 50 mg/kg in the haplo-HSCT setting^11–13^. Biju G et al. considered that PTCy at 50 mg/kg as the sole GVHD prophylaxis was practical in patients with SAA undergoing matched sibling donor HSCT (MSD-HSCT)^14^. Then, a study revealed that a lower dose of 40 mg/kg was available and safe as aGVHD prophylaxis in MMUD-HSCT as an alternative to ATG^15^. Nevertheless, patients with SAA in the allo-HSCT setting usually are at greater risk of cardiotoxicity due to duplicate adverse effect of cyclophosphamide (CY) in the conditioning regimen and PTCy, and assessments of lower PTCy doses are lacking up. Herein, we evaluated the effectiveness and safety of a reduced-dose PTCy protocol combined with cotransplantation of PBSCs and UC-MSCs for patients with SAA.

## Results

### Patients

**Figure 1:**
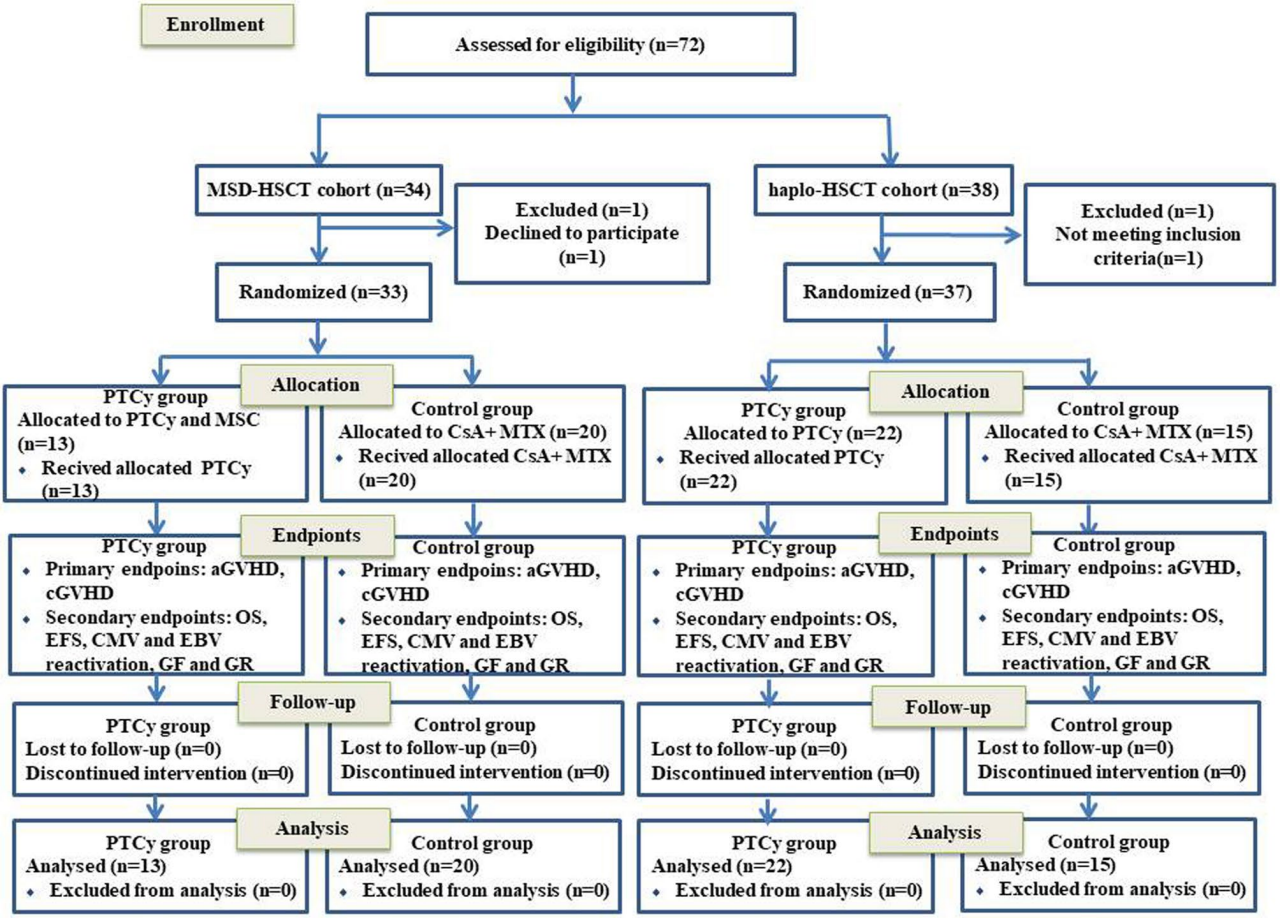
The flow diagram to describe the clinical course of patients.

33 patients who underwent MSD-HSCT and 37 patients who underwent haplo-HSCT were included and completed follow-up. There were 13 and 20 patients in MSD-PTCy and MSD-control groups, respectively, while there were 22 and 15 patients in MSD-PTCy and MSD-control groups, respectively. The flow diagram to describe the clinical course of patients was shown in Fig. 1. The clinical characteristics of patients in the MSD-HSCT and haplo-HSCT cohorts are summarized in Tables 1 and 2. No significant difference was observed between the PTCy cohorts and the control cohorts in terms of baseline demographics except for stem cell source. Eight patients (40%) of MSD-control cohort and all patients of haplo-control cohort received PBSCs and BM, while all patients of PTCy cohorts received PBSCs and UC-MSCs, except for two patients who suffered from primary graft failure received extra BM from a secondary donor in the haplo-PTCy cohort. There were 6 males (46.2%) and 13 males (59.1%) in the MSD-PTCy and haplo-PTCy cohorts and the median ages were 20 (range 9 to 35) and 14 (range 3 to 31) years respectively. One patient of each PTCy cohort was treated with anti-human lymphocyte immunoglobulin pretransplantation. The remaining patients had previously received treatment with cyclosporine (CSA) and/or steroids. In addition, two patients (9.1%) had hospital admissions for pulmonary infection requiring treatments prior to admission in the haplo-PTCy cohort, and one of the two patients had additional fungal sinus infections. Fortunately, the infections of the two patients were contained after engraftment.

**Table 1:** Patient characteristics and transplant-related parameters in the MSD-HSCT groups.

Variable	MSD-PTCy group (n = 13)	MSD-control group (n = 20)	*P* values
**Median age in years (range)**	20 (9–35)	22 (4–37)	0.882
**Sex(male/female)**	6/7	13/7	0.472
**Classification**			
SAA-I	8	13	
SAA-II	5	7	1.000
**Prior treatment**			
Steroids/cyclosporine	12	19	
ATG/ALG	1	1	1.000
**Time from diagnosis to HSCT**			
median months (range)	3 (1–63)	4 (1–84)	0.743
Median donor age in Years (range)	24 (4–45)	15 (4–37)	0.849
**Donor/recipient sex**			
Female/female	2	3	
Female/male	5	5	
Male/female	5	4	
Male/male	1	8	0.215
**ABO incompatibility**			
None	6	10	
Minor	1	5	
Major	6	5	0.304
**Graft source**			
PBSCs	13	12	
PBSCs and BM	0	8	0.012*
UC-MSCs	13	0	< 0.0001****
**Cell dose (median)**			
MNCs × 10^8^/kg	16.36 (12.57–23.91)	14.04 (6.33–24.95)	0.060
CD34 + cells × 10^6^/kg	7.39 (4.35–18.4)	5.39 (1.02–13.37)	0.082

Continuous variables were expressed as median values and ranges; Discrete variables were expressed as counts (%). *P*-value from Fisher exact test and independent samples T test.

*P < 0.05, ****P < 0.0001.

**Table 2:** Patient characteristics and transplant-related parameters in the haplo-HSCT groups.

Variable	Haplo-PTCy group (n = 22)	Haplo-control group (n = 15)	*P* values
**Median age in years (range)**	14 (3–31)	8 (2–32)	0.277
**Sex(male/female)**	13/9	9/6	1.000
**Classification**			
SAA-I	13	9	
SAA-II	9	6	1.000
**Prior treatment**			
Steroids/cyclosporine	21	14	
ATG/ALG	1	1	1.000
**Time from diagnosis to HSCT**			
median months (range)	13.5 (1–102)	7 (1–36)	0.028*
Median donor age in Years (range)	30 (4–53)	30 (17–53)	0.377
**Donor/recipient sex**			
Female/female	3	1	
Female/male	3	1	
Male/female	6	5	
Male/male	10	8	0.795
**HLA mismatch class**			
Class I mismatch (HLA A, B, C)	16	11	
Class II mismatch (HLA DRB1, DQB1)	6	4	1.000
**ABO incompatibility**			
None	12	10	
Minor	4	2	
Major	6	3	0.762
**Graft source**			
PBSCs	20	0	
PBSCs and BM	2	15	< 0.0001****
UC-MSCs	22	0	< 0.0001****
Donor-specific antibody	2	0	0.505
**Cell dose (median)**			
MNCs × 10^8^/kg	18.73 (8.41–32.35)	23.43 (2.02–31.72)	0.308
CD34 + cells × 10^6^/kg	7.60 (4.21–19.45)	8.95 (1.76–22.53)	0.522

Continuous variables were expressed as median values and ranges; Discrete variables were expressed as counts (%). *P*-value from Fisher exact test and independent samples T test.

*P < 0.05, ****P < 0.0001.

### Engraftment

In the MSD-PTCy cohort, all patients successfully engrafted. The median numbers of infused MNCs and CD34 + cells between the MSD-PTCy and control cohorts were similar (MNCs: 16.36 (range 12.57 to 23.91) × 10^8^/kg vs 14.04 (range 6.33 to 24.95) × 10^8^/kg; CD34 + cells: 7.39 (range 4.35 to 18.4) × 10^6^/kg vs 5.39 (range 1.02 to 13.37) × 10^6^/kg). However, we observed that the times to neutrophil and platelet engraftment were shorter in the MSD-PTCy cohort than those in the control cohort [11 days (range 10 to 14) vs 13 days (range 10 to 23) and 12 days (range 9 to 15) vs 14 days (range 10 to 45)]. The difference in graft rejection between the MSD-PTCy and control cohorts was not significant (7.7% vs 15.0%). The rates of chimerism at day + 30 in the MSD-PTCy and control cohorts were 100% and 90% respectively, and there was no significant difference.

In the haplo-HSCT cohorts, the median numbers of infused MNCs and CD34+ cells were comparative (MNCs: 18.73 (8.41–32.35) × 10^8^/kg vs 23.43 (2.02–31.72) × 10^8^/kg; CD34 + cells: 7.60 (4.21–19.45) × 10^6^/kg vs 8.95 (1.76–22.53) × 10^6^/kg). Two patients suffered from primary graft failure experienced successful engraftment after receiving transplant from haploidentical donors in the haplo-PTCy cohort. In the haplo-control cohort, secondary graft failure developed in one patient at 1.5 months after transplantation, and this patient sustained engraftment after undergoing MMUD-HSCT. The times to neutrophil and platelet engraftment were shorter in the haplo-PTCy cohort than those in the control cohort [12 days (range 9 to 15) vs 13 days (range 11 to 19) and 11.5 days (range 9 to 17) vs 14 days (range 11 to 62)]. One patient from each cohort suffered graft rejection.

### Acute and chronic GVHD

**Figure 2:**
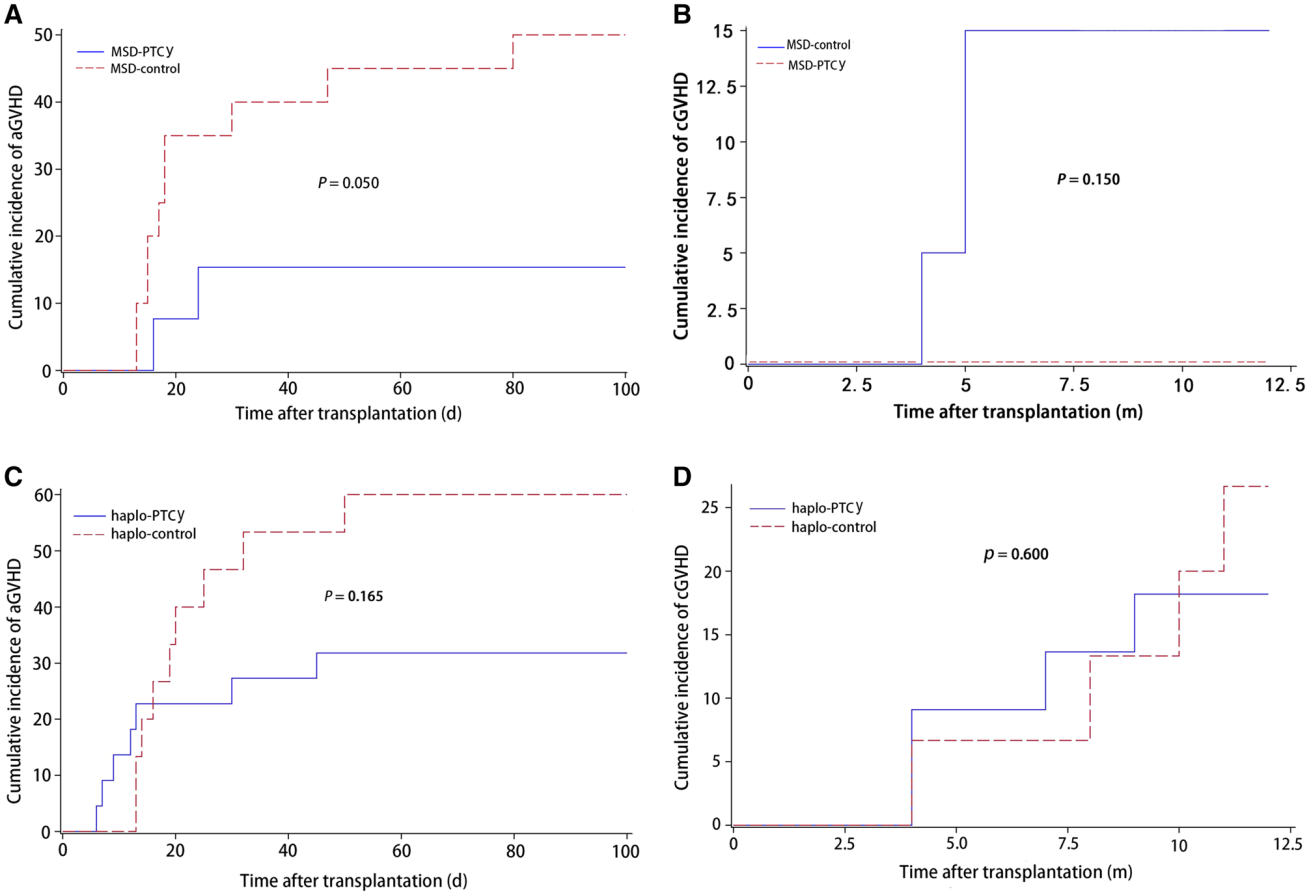
Cumulative incidence of graft-versus-host disease (GVHD). (**A**) Cumulative incidence of acute GVHD at day + 100 in MSD-PTCy group compared to MSD-control group. (**B**) Cumulative incidence of chronic GVHD at 1 year in MSD-PTCy group compared to MSD-control group. (**C**) Cumulative incidence of acute GVHD at day + 100 in haplo-HSCT group compared to haplo-control group. (**D**) Cumulative incidence of chronic GVHD at 1 year in haplo-HSCT group compared to haplo-control group.

The cumulative incidence of aGVHD at day + 100 in the MSD-PTCy cohort was 15.4% (95% CI 2.2–39.9) compared to 50.0% (95% CI 26.3–69.8) in the control cohort (*P* = 0.050) (Fig. 2A). The cumulative incidence of grade II-IV aGVHD in the MSD-PTCy cohort was lower than that in the control cohort, 7.7% (95% CI 0.4–30.3) vs 30.0% (95% CI 11.8–50.7), but the difference did not reach statistical significance (*P* = 0.122) (Table 3). No patient developed cGVHD in the MSD-PTCy cohort. In contrast, the 1-year cumulative incidence of cGVHD in the control cohort was 15.0% (95% CI 3.5–34.1) (Fig. 2B).

**Table 3:** Transplantation outcomes in MSD-HSCT groups.

Variable	MSD-PTCy group (n = 13)	MSD-control group (n = 20)	*P* values
**Time to ANC recovery (median)**	11 (10–14)	13 (10–23)	0.014*
**Time to platelets recovery (median)**	12 (9–15)	14 (10–45)	0.027*
**Chimerism at day + 30 (n, %)**			
Full chimerism	13 (100)	18 (90.0)	0.508
Graft rejection (n, %)	1 (7.7)	3 (15.0)	1.000
aGVHD cumulative incidence % (95% CI)	15.4 (2.2–39.9)	50.0 (26.3–69.8)	0.050*
Grade II–IV aGVHD cumulative incidence % (95% CI)	7.7 (0.4–30.3)	30.0 (11.8–50.7)	0.122
cGVHD 1-year cumulative incidence % (95% CI)	0	15.0 (3.5–34.1)	0.150
**Complications (n, %)**			
Pulmonary infection	1 (7.7)	10 (50.0)	0.022*
CMV	3 (23.1)	13 (65.0)	0.032*
EBV	0	12(60.0)	0.001***
Hemorrhagic cystitis	1 (7.7)	7 (35.0)	0.108
1-year OS % (95% CI)	100	75.0 (50.0–88.7)	0.057
1-year EFS % (95% CI)	92.3 (56.6–98.9)	65.0 (40.3–81.5)	0.071
Follow-up, months from infusion, median	22 (10–36)	21.5 (2–36)	0.995

95% CI 95% confdence interval. Continuous variables were expressed as median values and ranges; Discrete variables were expressed as counts (%). Cumulative incidences of aGVHD and cGVHD were estimated using competing risk model. *P*-value log-rank tests and Fisher exact test.

*P < 0.05, ***P < 0.001.

The cumulative incidence of aGVHD at day + 100 in the haplo-PTCy cohort was lower than that in the control cohort (Table 4), 31.8% (95% CI 13.8–51.6) vs 60.0% (95% CI 29.9–80.6), but we observed no significant difference (*P* = 0.165) (Fig. 2C). The cumulative incidence of grade II–IV aGVHD in the haplo-PTCy cohort was 27.3% (95% CI 10.8–46.9) compared to 53.3% (95% CI 24.8–75.3) in the control cohort(*P* = 0.170). Notably, one patient with grade IV liver aGVHD had intracranial aGVHD, and one patient with aGVHD developed skin cGVHD in the haplo-PTCy cohort. The 1-year cumulative incidences of cGVHD between the haplo-HSCT cohorts were no difference (*P* = 0.600), 18.2% (95% CI 5.5–36.8) vs 26.7% (95% CI 7.7–50.7) (Fig. 2D). There was no extensive cGVHD in the haplo-PTCy cohort, while one patient developed extensive cGVHD in the control cohort.

**Table 4:** Transplantation outcomes in haplo-HSCT groups.

Variable	Haplo-PTCy group (n = 22)	Haplo-control group (n = 15)	*P* values
**Time to ANC recovery (median)**	12 (9–15)	13 (11–19)	0.013*
**Time to platelets recovery (median)**	11.5 (9–17)	14 (11–62)	0.045*
**Chimerism at day + 30 (n, %)**			
Full chimerism	22 (100)	14 (93.3)	0.405
Graft rejection (n,%)	1 (4.5)	1 (6.7)	1.000
aGVHD cumulative incidence % (95% CI)	31.8 (13.8–51.6)	60.0 (29.9–80.6)	0.165
Grade II–IV aGVHD cumulative incidence % (95%CI)	27.3 (10.8–46.9)	53.3 (24.8–75.3)	0.170
cGVHD 1-year cumulative incidence % (95% CI)	18.2 (5.5–36.8)	26.7 (7.7–50.7)	0.600
**Complications (n, %)**			
Pulmonary infection	9 (40.1)	9 (60.0)	0.325
Mild to moderate/severe (n)	7/2	7/2	1.000
CMV	18 (81.8)	8 (53.3)	0.080
EBV	5 (22.7)	12 (80.0)	0.001***
Hemorrhagic cystitis	7 (31.8)	4 (26.7)	1.000
1-year OS % (95% CI)	81.8 (58.5–92.8)	77.1 (44.2–92.1)	0.890
1-year EFS % (95% CI)	66.9 (39.1–84.2)	48.0 (20.5–71.2)	0.404
Follow-up, months from infusion, median	11.5 (3–27)	12 (1.5–36)	0.681

Continuous variables were expressed as median values and ranges; Discrete variables were expressed as counts (%). Cumulative incidences of aGVHD and cGVHD were estimated using competing risk model. *P*-value log-rank tests and Fisher exact test.

*P < 0.05, ***P < 0.001.

### Regimen-related toxicities

The incidence of pulmonary infection in the MSD-PTCy cohort was obviously lower than that in the control cohort (*P* = 0.022), 7.7% vs 50.0%. Similarly, the incidences of Cytomegalovirus (CMV) and Epstein-Barr virus (EBV) reactivation in the MSD-PTCy cohort were significantly lower compared with those in the control cohort (CMV: 23.1% vs 65.0%; EBV: 0 vs 60.0%), and there were significant differences between the two cohorts (*P*: 0.032 and 0.001, respectively). Although the incidence of hemorrhagic cystitis was lower in the MSD-PTCy cohort (7.7% vs 35.0%), the difference was not significant (*P* = 0.108). One patient suffered from pure red cell aplasia (PRCA) after ABO-compatible HSCT, and hematopoietic recovery occurred eighteen months after transplantation. In the control cohort, four of twelve patients with EBV reactivation developed posttransplantation lymphoproliferative disorders (PTLD) and three patients were cured through combination therapy with immunosuppressor withdrawal, rituximab and donor lymphocyte infusion (DLI). Another patient experienced an epileptic seizure because of intracranial EBV infection. Furthermore, two patients developed unexplained seizures that were relieved after expectant treatment. Cardiotoxicity and thrombotic microangiopathy (TMA) each developed in one patient. No patient had veno-occlusive disease (VOD).

The incidence of pulmonary infection in the haplo-PTCy cohort was lower than that in the control cohort (40.1% vs 60.0%), while the incidence of CMV reactivation in the haplo-HSCT cohort was higher compared to that in the control cohort (81.8% vs 53.3%), but the differences were not statistically significant (*P*: 0.325, 0.080). The median times to CMV infection were + 37 days (range 29 to 46) and + 35 days (range 25 to 53) in the haplo-HSCT and the control cohorts respectively. Two of eighteen patients with CMV infection in the haplo-HSCT cohort and one of eight patients with CMV infection in the control cohort developed cytomegalovirus retinitis. The incidence of EBV reactivation in the haplo-HSCT cohort (22.7%) was obviously lower than that in the control cohort (80.0%), and the difference was significant (*P* = 0.001). There were no PTLD in the haplo-PTCy cohort, but three patients developed PTLD based on their seropositive EBV status in the control cohort. The incidences of hemorrhagic cystitis with BKV seropositivity were comparative between the haplo-HSCT and the control cohorts (31.8% and 26.7%). In the haplo-PTCy cohort, one of seven patients who were seropositive for BKV progressed to BKV encephalitis and suffered from intracranial fungal infection. In addition, one patient had TMA at day + 50. Moreover, posterior reversible encephalopathy syndrome (PRES) developed in four patients in the haplo-PTCy cohort and in two patients in the control cohort. None of all patients developed VOD in the two cohorts.

### Survival

The median follow-up time of the MSD-PTCy cohort was similar to that in the control cohort, 22 months (range 10 to 36) vs 21.5 months (range 2 to 36). The median follow-up of the haplo-HSCT and the control cohorts were 11.5 months (range 3 to 27) and 12 months (range 1.5 to 36), respectively.

**Figure 3:**
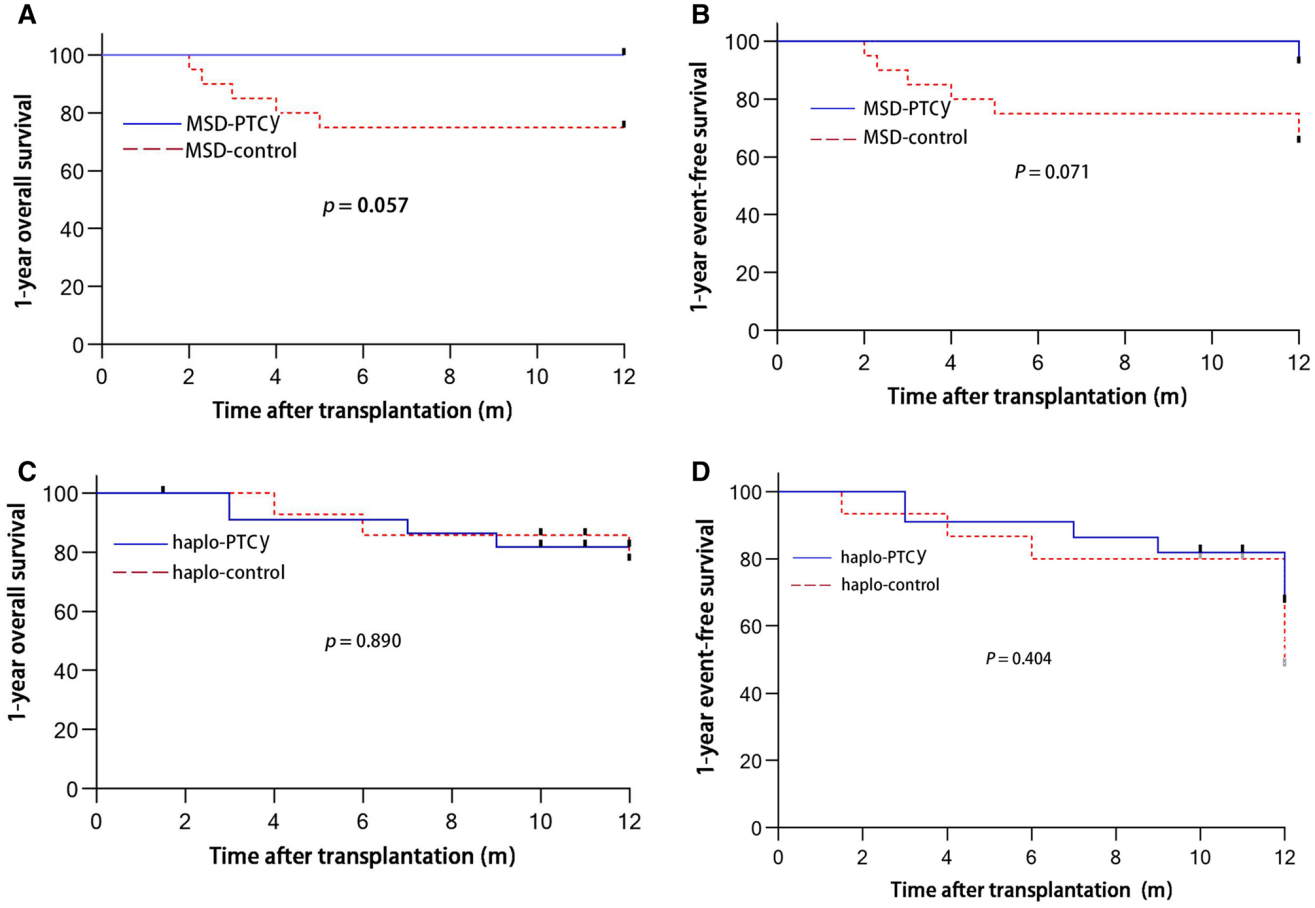
Kaplan–Meier curves for overall survival (OS) and event-free survival (EFS). (**A**) Cumulative percentage of 1-year OS in MSD-PTCy group compared to MSD-control group. (**B**) Cumulative percentage of 1-year EFS in MSD-PTCy group compared to MSD-control group. (**C**) Cumulative percentage of 1-year OS in the haplo-HSCT group compared to haplo-control group. (**D**) Cumulative percentage of 1-year EFS in the haplo-HSCT group compared to haplo-control group.

All thirteen (100%) patients in the MSD-PTCy cohort are now alive with normal hematopoiesis. Interestingly, one female is now three months pregnant. The 1-year overall survival (OS) of the MSD-PTCy cohort was 100% compared to 75.0% (95% CI 50.0–88.7)% in the MSD-control cohort (*P* = 0.057) (Fig. 3A). The 1-year event-free survival (EFS) of the MSD-PTCy cohort was 92.3% (95% CI 56.6–98.9)% compared to 65.0% (95% CI 40.3–81.5)% in the MSD-control cohort (*P* = 0.071) (Fig. 3B). In the control cohort, three patients died from serious pulmonary infection at 2, 3 and 5 months, one patient died of intracranial EBV infection at day + 70, and one patient died of TMA at 4 months. In the MSD-PTCy cohort, twelve of thirteen patients had a follow-up period of more than 12 months at the time of writing. All the patients began to reduce immunosuppressor treatment at 6 months and these agents were withdrawn before 9 months except for the patient with PRCA.

In the haplo-PTCy cohort, two patients died from intractable pulmonary infection at 3 and 9 months, one patient died of cerebral hemorrhage at 3 months, and one patient died of intestinal GVHD at 7 months. In the control cohort, two patients died from pulmonary infection at 4 and 6 months, and one patient died of severe intestinal GVHD at 12 months. The cumulative incidence of 1-year OS was 81.8% (95% CI 58.5–92.8) in the haplo-PTCy cohort compared to 77.1% (95% CI 44.2–92.1) in the haplo-control cohort (Fig. 3C). The 1-year EFS in the haplo-PTCy cohort vs the control cohort was 66.9% (95% CI 39.1–84.2) vs 40.8% (95% CI 20.5–71.2) (Fig. 3D). However, there was no significant difference in 1-year OS or EFS between two cohorts. In the haplo-PTCy cohort, eleven of eighteen living patients had a follow-up period of more than 12 months at the time of writing, those patients began to reduce immunosuppressor treatment at 9 months after HSCT.

## Discussion

Effective therapies for SAA patients usually involve immunosuppressive therapy (IST), allo-HSCT and eltrombopag. The application of IST is limited by late sequelae and the high cost of ATG^16^. Although the effective rate of eltrombopag is 20% to 40%, the main problems are relapse and secondary clonal disease^17^. The key challenge of allo-HSCT lies in minimizing complications and maximizing survival. Recently, PTCy has gradually come to be the predominate treatment worldwide due to its favorable prognosis in the allo-HSCT setting^18,19^. However, a high dose of PTCy is accompanied by cardiotoxicity. Therefore, some studies have reported the outcomes of reduced doses of PTCy in malignant and nonmalignant hematological disorders^15,20^. Furthermore, UC-MSCs are popular in allo-HSCT for SAA because of several beneficial effects, including facilitating engraftment and decreasing GVHD^21,22^. At present, there are few evaluations of reduced-dose PTCy for SAA patients receiving cotransplantation of PBSCs and UC-MSCs. Therefore, the aim of this prospective study was to demonstrate the influence of reduced-dose PTCy on early outcomes after cotransplantation of UC-MSCs and PBSCs for patients with SAA receiving transplants from either matched or haploidentical donors.

GVHD is a main barrier in patients with SAA undergoing allo-HSCT. PTCy, an attractive option, eliminates early proliferating alloreactive T cells to reduce impaired immune reconstitution^23,24^. However, a high dose of PTCy is accompanied by cardiotoxicity. Then, the doses of PTCy were reduced to 20 mg/kg and 25 mg/kg from the conventional 50 mg/kg dose for MSD-HSCT and haplo-HSCT, respectively. The results showed that the cumulative incidence of aGVHD at day + 100 in the MSD-PTCy cohort was lower compared to that in the control cohort(*P* = 0.050). In addition, the cumulative incidence of grade II–IV aGVHD was lower in the MSD-PTCy cohort than that in the control cohort, 7.7% (95% CI 0.4–30.3) vs 30.0% (95% CI 11.8–50.7), but we did not observe a significant difference. No patient in the MSD-PTCy cohort developed cGVHD. George et al. reported that 30 patients with SAA received high-dose PTCy as the sole GVHD prophylaxis in PBSC MSD-HSCT. The incidences of grade II-IV aGVHD and cGVHD were 22.0% and 22.7%, respectively^14^. In MSD-HSCT for malignant hematologic disorders, high-dose PTCy has been associated with the occurrence of grade II-IV aGVHD (17% to 45%) and cGVHD (7% to 14%)^25–27^. A multicenter study showed that the cumulative incidences of grade II–IV aGVHD and cGVHD at 1 year were 33.7% and 22.4%, respectively, for SAA patients receiving BM and PBSCs in the haplo-HSCT setting^28^. In this study, the cumulative incidence of grade II-IV aGVHD at day + 100 and 1-year cumulative incidence of cGVHD in the haplo-PTCy cohort were 27.3% (95% CI 10.8–46.9) and 18.2% (95% CI 5.5–36.8), respectively. Although there were no significant differences in either the cumulative incidence of aGVHD and grade II–IV aGVHD or the 1-year cumulative incidence of cGVHD, reduced-dose PTCy and cotransplantation of PBSCs and UC-MSCs probably overcame the high incidences of aGVHD and cGVHD due to PBSCs. Encouragingly, the 1-year OS in the MSD-PTCy cohort was 100%. We observed no difference in the 1-year OS and EFS values between the haplo-PTCy and haplo-control cohorts, but we believe that the joint use of PBSCs and UC-MSCs leads to decreased donor pain and improves donation rates compared to the use of routine PBSCs and BM.

The times to neutrophil and platelet engraftment in the PTCy cohorts were shorter than those in the control cohorts (*P* < 0.05). The time to neutrophil and platelet engraftment in the haplo-PTCy cohort were 12 days (9–15) and 11.5 days (9–17), respectively. Moreover, the times to engraftment in the haplo-HSCT cohort were heterogeneous, with the time to neutrophil engraftment being 12 to 19 days, and the time to platelet engraftment being 13 to 21 days^28–31^. It followed that the time to engraftment was not delayed due to reduced-dose PTCy. The application of UC-MSCs was also special in this study. Various abilities of MSCs in the HSCT setting involve facilitating engraftment, remedying graft dysfunction and providing GVHD prophylaxis^32^. A retrospective study demonstrated that UC-MSCs were likely to decrease the occurrence of severe aGVHD, which influenced life quality and prognosis for patients undergoing haplo-HSCT^33^. Another study reached the same conclusion and verified that cotransplantation of UC-MSCs with haplo-HSCT was feasible and safe for patients with SAA^34^. Platelet-derived growth factor (PDGF) secreted from MSCs, a putative anti-apoptotic factor, shows potential capability to stimulate platelet recovery^35,36^. There is controversy regarding the engraftment function of MSCs, but most studies have shown that the role of MSCs in allo-HSCT is worthy of consideration^33,34,37^. However, there are some limitations of adult BM-MSCs, such as quality variation from different donors, limited proliferative potency and stem cell senescence. Same pluripotent stem cells (PSC)-derived MSCs (PSC-MSCs) with batch-to-batch consistency are another alternative for quality control. PSC-MSCs present higher proliferative potential^38^. A phase I clinical trail demonstrated the potential capability of GMP-grade PSC-MSCs in refractory GVHD^39^. Then, PSC-MSCs may provide another putative cellular source overcome many limitations of adult BM-MSCs.

The incidence of viral infections is another problem for allo-HSCT protocols using PTCy. In this study, the viral infection rates were lower in the MSD-PTCy cohort than those in the control cohort, both for CMV and EBV reactivation (*P* < 0.05). It is worth noting that no patient underwent EBV reactivation in the MSD-PTCy cohort. Patients with CMV reactivation especially have a risk for developing CMV-related diseases, including CMV pneumonia, retinitis and enteritis. Two patients and one patient with CMV seropositivity in the haplo-PTCy and haplo-control cohorts developed CMV retinitis, respectively. As a result, they developed varying degrees of visual impairment. However, the incidence of CMV reactivation in the haplo-HSCT cohort was higher than that in the haplo-control cohort, although there was no significant difference, which may be due to the additive immunosuppressive effects of ATG and PTCy. How to reduce the incidence of CMV reactivation is an outstanding problem. In the haplo-HSCT cohort, the incidence of EBV reactivation was significantly lower than that in the haplo-control cohort (*P* < 0.05), which was attributed to the use of rituximab at day + 5. Bacigalupo et al. showed that patients received rituximab at day + 5 after alternative donor HSCT had significantly lower rates of EBV-DNA and lower maximum median EBV copies than the control cohort (*P* < 0.05). Furthemore, rituximab at day + 5 had no influence on incidences of infections^40^. Pulmonary infection is one of the key life-threatening complications. The incidence of pulmonary infection in the MSD-PTCy cohort was obviously lower than that in the control cohort (*P* < 0.05). It is worth mentioning that the total dose of ATG was reduced from 10 to 8 mg/kg in the MSD-PTCy cohort. Though no difference, the incidence of pulmonary infection in the haplo-PTCy cohort was lower than that in the haplo-control cohort, 40.1% vs 60.0%.

There are some limitations in the study. First, the small sample size and short follow-up period were weaknesses. Thus, a prospective study with a large sample size is necessary. In addition, further studies are required to evaluate the clinical utility of reduced-dose PTCy and UC-MSCs in preventing and alleviating GVHD, promoting engraftment and improving outcome. Despite these limitations, the protocol does not increase the high incidence of aGVHD and cGVHD due to PBSCs without compromising efficacy of MSD-HSCT and haplo-HSCT, and also decreases difficulties for donors. In conclusion, it is feasible for patients with SAA to receive reduced-dose PTCy and cotransplantation of PBSCs and UC-MSCs.

## Methods

### Study design and patients

The study aim was to analyze patients with SAA who underwent MSD-HSCT and haplo-HSCT with cotransplantation of PBSCs and UC-MSCs at the Department of Hematology, Affiliated Cancer Hospital of Zhengzhou University from March 2017 to June 2019. We compared the engraftment time, cumulative incidences of aGVHD and cGVHD, incidence of infection and outcome (1-year EFS and 1-year OS) in our MSD-PTCy cohort and haplo-PTCy cohort with those contemporary in a cohort of 20 patients and in a cohort of 15 patients receiving CSA and short-course methotrexate (MTX) as GVHD prophylaxis, respectively. The study was approved by the Institutional Review Board (IRB) of Henan Cancer Hospital and registered in the Chinese Clinical Trial Registry (ChiCTR1900026462, 11/10/2019). All patients and/or guardians signed informed consent forms approved by the IRB of Henan Cancer Hospital (2019287). All experiments and methods performed in the study were in accordance with relevant guidelines and regulations. In this study, 35 patients with SAA were administrated with PTCy and cotransplantation of PBSCs and UC-MSCs, including 13 patients who underwent MSD-HSCT and 22 patients who underwent haplo-HSCT. Furthermore, all patients with SAA met the inclusion/ exclusion criteria: (1) voluntary participation in allo-HSCT; (2) absence of systemic active infections and severe heart, liver, kidney dysfunction; (3) Eastern Cooperative Oncology Group score was no more than 2. Patients with heart, liver, or kidney dysfunction were excluded. Written informed consent was obtained from patients or their guardians. To address potential sources of bias, patients who met the standards were randomly divided into PTCy group and control group in MSD-HSCT and haplo-HSCT cohorts.

### Transplantation procedure

Thirteen patients in the MSD-HSCT cohort received a conditioning regimen consisting of fludarabine (FLU) 30 mg/m^2^/day for 5 days given on days − 6 to − 2, ATG 2 mg/kg/day for 4 days given on days − 4 to − 1, and CY 24 mg/kg/day for 5 days given on days − 6 to − 2. Twenty-two patients in the haplo-HSCT cohort received a conditioning regimen consisting of FLU 30 mg/m^2^/day for 5 days given on days − 6 to − 2, ATG 2.5 mg/kg/day for 4 days given on days − 4 to − 1, CY 24 mg/kg/day for 5 days given on days − 6 to − 2, and a single 2.5 ~ 3 Gy of total body irradiation (TBI) on day − 1. Two patients who underwent haplo-HSCT without TBI suffered graft failure and underwent secondary transplantation. The second conditioning regimen included busulfan 0.8 mg/kg, q6h on days − 3 to − 2, and 3 Gy TBI on day − 1. CSA was administered with from day + 5 and PTCy 20 mg/kg was administered on days + 3 and + 4 for patients in the MSD-PTCy cohort. CSA combined with mycophenolate (MMF) was administered from day + 5 and PTCy 25 mg/kg was administered on days + 3 and + 4 for patients in the haplo-PTCy cohort. The consecutive patients in the control cohorts received short-course MTX at a dose of 10 mg/m^2^ on day + 1 followed by a dose of 7 mg/m^2^ on days + 3, + 6 and + 11, with CSA and/or MMF used as GVHD prophylaxis. The graft sources for all patients were PBSCs and MSCs in the PTCy cohorts. Intravenous infusions of UC-MSCs were given to all patients with PTCy on day 0 at a dose of 1 × 10^7^/kg.

### Supportive care

Patients were hospitalized in a laminar flow ward with positive pressure and high-efficiency particulate air filtration until neutrophil recovery. Standard infection prophylaxis with a quinolone or berberine, trimethoprim-sulfamethoxazole and nystatin was begun on day − 7. Standard Pneumocystis jiroveci prophylaxis was given to patients after engraftment for 1 year. Patients received ganciclovir at 5 mg/kg in the conditioning period. Broad-spectrum antibiotics, antifungals were used for agranulocytosis or fevers. Granulocyte colony-stimulating factor (G-CSF) was given intravenously starting on day + 5 at 5 µg/kg/day until the peripheral blood absolute neutrophil count (ANC) was ≥ 0.5 × 10^9^/L for 3 days. Rituximab was given as prophylaxis for EBV on day + 5 in the haplo-PTCy cohort. Blood products were irradiated with 25 Gy. CMV reactivation was monitored by quantitative PCR twice a week until at least day + 100. Preemptive therapy with ganciclovir or foscarnet was applied. EBV was also monitored weekly by quantitative PCR.

### Posttransplantation complications

Post-HSCT complications mainly involve aGVHD, cGVHD, infections and other toxicities. GVHD was graded in accordance with the consensus criteria for aGVHD^41^ and cGVHD^42^. The diagnosis of GVHD depended on clinical symptoms and/or skin, oral mucosa, liver and gut biopsies^42–44^. The grade of nonhematologic and noninfectious toxicity was diagnosed according to the Common Terminology Criteria for Adverse Events (CTCAE, v4.0) of the US National Cancer Institute and National Institutes of Health from day 0 to day + 360.

### Engraftment and chimerism

Neutrophil engraftment was defined as the first of three consecutive days with an absolute ANC ≥ 0.5 × 10^9^/L. Platelet engraftment was defined as the first day of seven consecutive days with a platelet count ≥ 20 × 10^9^/L. Primary graft failure was defined as failure to achieve an ANC of 0.5 × 10^9^/L by day + 28. Secondary graft failure was defined as an ANC < 0.5 × 10^9^/L after initial engraftment that was independent of infection, medications and insensitive to G-CSF. Full donor chimerism was defined as ≥ 95% leukocytes of donor origin in peripheral blood and/or bone marrow samples^45^. Graft rejection was defined as a progressive decrease in blood counts after initial engraftment with loss of donor chimerism.

### Statistical analysis

Median values and ranges were expressed for continuous variables and percentages for categorical variables. Continuous variables were analyzed by an independent samples T test. Differences in categorical variables were compared by Fisher’s exact test. Cumulative incidences of aGVHD and cGVHD were estimated using competing risk model with early death being a competing event. OS and EFS were performed with the Kaplan–Meier method and log-rank tests, while median survival was estimated with a 95% confidence interval. A two-sided *P*-values less than 0.05 were considered statistically significant. Analyses were performed using GraphPad Prism for Windows, version 8.4.1 and SAS version 9.4 (SAS Institute, Cary, NC).

## Data Availability

The datasets generated during and/or analysed during the current study are available from the corresponding author on reasonable request.
